# Effectiveness of TCM cauterization in recurrent tonsillitis: A protocol for systematic review and meta-analysis

**DOI:** 10.1097/MD.0000000000022597

**Published:** 2020-10-09

**Authors:** Sha Li, Hui Xie, Han-Jen Chiang, Zhiqing Liu, Zhenzhen Han, Jiao Liang, Lu Wang, Qiu Wang, Jiongke Li, Yusi Li

**Affiliations:** a Hospital of Chengdu University of Traditional Chinese Medicine, Chengdu; b Yibin Hospital of T.C.M, West of South Bank District, Yibin City, Sichuan Province, P.R. China.

**Keywords:** cauterization, meta-analysis, protocol, recurrent tonsillitis

## Abstract

**Introduction:**

Recurrent tonsillitis (RT) is often treated with antibiotic therapy and surgery. Although these treatments have advantages, they are also controversial. The purpose of this study is to analyze the safety and effectiveness of traditional Chinese medicine (TCM) cauterization in the treatment of RT, so as to provide an alternative for the clinicians and to cover the shortage of therapeutic methods.

**Methods and analysis:**

This protocol is guided by the Preferred Reporting Items for Systematic Review and Meta-Analysis Protocols (PRISMA-P) and by the Cochrane Collaboration Handbook. We will formulate strict inclusion and exclusion criteria in English databases (PubMed, EMBASE, and Web of Science), Chinese databases (CNKI, Wanfang databases, CBM, and VIP), and search literatures in different clinical registration platforms (Cochrane Library, Chinese Cochrane Centre's Clinical Trial Registry Platform). The included articles will be evaluated using Cochrane RCT evaluation criteria. Stata 15.0 will be used for data analysis. Subgroup analysis, sensitivity analysis, and meta-regression will detect sources of heterogeneity. Egger's Test or Begg's Test will detect publication bias quantitatively.

**Conclusion:**

Cauterization can effectively control the recurrence of tonsillitis through clinical trials, but evidence-based medicine needs to be adopted to provide strong evidence for its effectiveness. The purpose of our research is to provide the evidence.

**OSF Registration number:**

DOI 10.17605/OSF.IO/PZ69Q.

## 1 Introduction

Recurrent tonsillitis is a global chronic respiratory infectious disease^[1]^; but it is not just a respiratory disease which can cause damage to multiple parts of the body, such as cardiovascular system,^[2]^ urinary system,^[3]^ arthrosis,^[4]^ etc. This puts a heavy burden on the patients, their families and the entire medical system, and seriously affects their quality of life.

Decreased immunity,^[5]^ low zinc and iron content in tonsil tissues,^[6]^ persistent bacterial biofilm,^[7]^ vitamin D deficiency,^[8]^ abnormal gene expression,^[9]^ and other factors may be the reasons for the recurrence of the disease and pathological mechanism. Due to the immature immune system and environmental factors, children are more susceptible to this disease.^[10]^ The incidence of this disease is relatively high. In Germany, more than 120,000 patients visit a doctor because of recurrent tonsillitis.^[11]^

Antibiotic therapy, tonsillectomy, and waiting for observation are the main methods of treatment of the disease.^[11,12]^ Antibiotic therapy is suitable for a small number of patients with acute streptococcal infection. The course of treatment is 5 to 7 days, but the risk of antibiotic resistance may follow.^[11]^ For those patients who are in the asymptomatic period of RT and have not reached the indications for surgery, the guideline does not give advice and can only wait for observation, which increases the risk of complications. At present, the global antibiotic abuse and antibiotic resistance make the treatment of this disease face huge challenges. Within a year, when tonsillitis had more than 6 episodes, the guidelines recommend tonsillectomy,^[12]^ but there is still uncertainty whether surgery can benefit patients.^[13,14]^ These disputes make doctors and patients face many risks when jointly formulating the best treatment plan, which may increase the medical burden and affect the quality of life.

In China, an appropriate technique of traditional Chinese medicine, cauterization, is widely used in treating asymptomatic stage of recurrent tonsillitis; which can effectively control the recurrence of tonsillitis.^[15]^ Cauterization has been promoted in different levels of the health care system for its highly efficient, operation is simple, and relatively safe.^[16,17]^ This method was first recorded in “Qian Jin Yi Fang” by Sun Simiao, a famous doctor in the Tang Dynasty. It has been applied for thousands of years and has been widely used in various clinical diseases, such as spontaneous epistaxis,^[18]^ acute pharyngitis,^[19]^ suppurative mastitis,^[20]^ etc. Cautery can increase the number of T cell subsets in patients when reducing the number of recurrences of tonsillitis^[21]^; however, the research scope is small, the sample size is small, and there are limitations. Evidence-based medicine needs to be adopted to provide strong evidence for its effectiveness.

The purpose of this meta-analysis is to provide some possible evidence for the effectiveness of TCM cauterization in the treatment of recurrent tonsillitis, so as to provide an alternative therapy plan for patients with asymptomatic RT and to compensate for the disadvantages of antibiotic therapy and tonsillectomy.

## 2 Methods

This meta-analysis has been registered on OSF (registration number: DOI 10.17605/OSF.IO/PZ69Q) and will be carried out under the guidance of Preferred Reporting Items for Systematic Review and Meta-Analysis Protocols (PRISMA-P) and the Cochrane Collaboration Handbook.

All authors jointly developed the search strategy, inclusion criteria, exclusion criteria, article quality evaluation method, data extraction, and analysis strategy.

### 2.1 Search strategy

The two authors will independently search English databases (PubMed, Embase, and Web of Science), Chinese databases (CNKI, Wanfang database, CBM, and VIP) and clinical registration platforms (Cochrane Library, Chinese Cochrane Centre's Clinical Trial Registry Platform). The search terms include Chinese terms, such as, Ru E, Yang E, Yin E, Zhuo, Lao, Shao, and Tang, and English terms, such as, Tonsillitis, Cautery, Cauterization, Tonsillitides, etc, and the search time is from the establishment of the database to May 22, 2020. Boolean algorithm is used as search formula to search full-text articles with subject terms and free words. The search formula is follows: (“Tonsillitis” OR “Tonsillitides” OR “Ru E” OR “Yang E” OR “Yin E”) AND (“Cautery” OR “Cauterization” OR “Cauterize” OR “Shao” OR “Zhuo” OR “Lao” OR “Tang”) AND (“trial” OR “randomized clinical trial” OR “randomized controlled trial” OR “clinical trial”).

### 2.2 Inclusion and exclusion criteria

Inclusion criteria:

1. The participants of the study are patients diagnosed as “recurrent tonsillitis” with enlarged tonsils; regardless of age, gender, and race.
2. The intervention of the experiment group is TCM cauterization. The specific steps are as follows. First, press the tongue with a tongue depressor to fully expose the tonsils. Second, a suitable size of a soldering iron is selected according to the size of the tonsils, dip the iron into sesame oil immediately after being heated to slightly red, then quickly touch the surface of the tonsils one to two second(s). When taking out the soldering iron, the cauterized surface would form a pseudo-membrane layer. At least create 3 areas of pseudo-membrane layer as one therapeutic amount.
3. The intervention of the control group is oral medication; which includes oral Chinese herbal medicine and oral western medication.
4. The main observation indicators include the effective rate of TCM cauterization, the change of tonsil size, and the recurrence rate of tonsillitis. Secondary observation indicators include the quality of life assessment and the number of adverse events (such as bleeding, infection, and complications).
5. The study type is a randomized controlled study.

Exclusion criteria:

1. Patients with acute attack of recurrent tonsillitis will be excluded.
2. Patients with recurrent tonsillitis accompanied with other symptoms will be excluded.
3. If the selected articles are abstracts, letters, case reports, reviews, or nonclinical studies, they will all be excluded.
4. If the experiment group is not a single-cautery study or the control group is not a single-drug treatment study, they will be excluded.
5. Articles that reuse data will be excluded.

### 2.3 Article retrieval process

**Figure 1:**
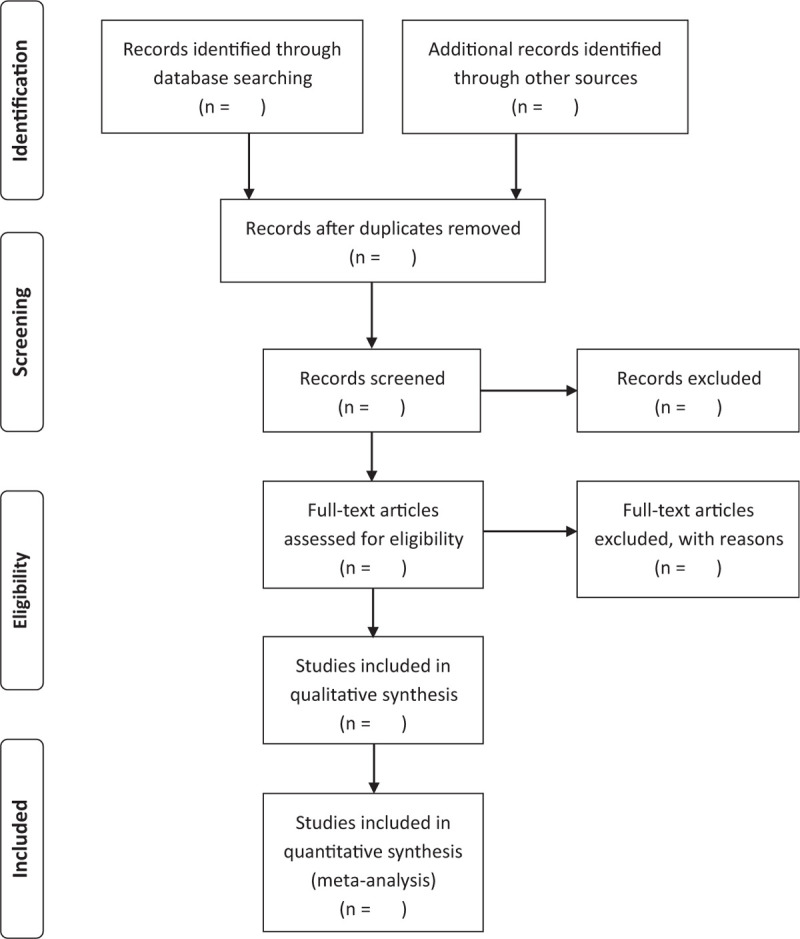
Flow diagram of the identification and selection studies.

Two authors will independently carry out a strict literature screening according to the flowchart shown below (Fig. 1) and all the selected articles will go through data analysis. If there is a dispute during the screening process, a third author will participate in the discussion and make the final decision on whether or not the article(s) will be included or excluded.

### 2.4 Data extraction

All candidate articles were evaluated and extracted by two independent authors. If disagreement occurred, two authors discussed and arrived at consensus with a third author. Information from each article will be recorded based on the following table in an Excel document; which includes first author, year of publication, country of publication, study design, total number of cases and gender, follow-ups, treatment strategy, control strategy, etc (Table 1).

**Table 1:**
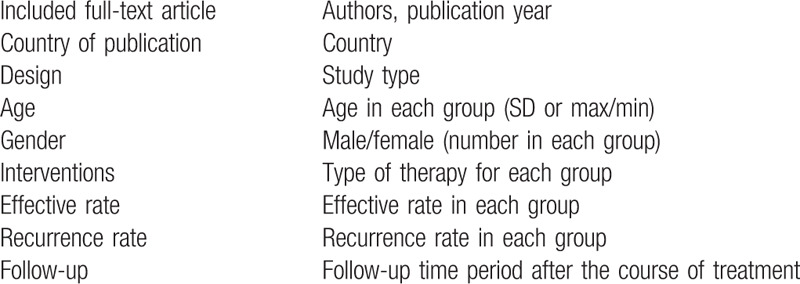
Main characteristics of the studies included in the meta-analysis.

### 2.5 Quality assessment of the studies

The quality of the articles included in the study will be assessed based on the Cochrane Collaboration Handbook. It contains six parts, namely: Sequence Generation, Allocation Concealment, Blinding, Incomplete outcome data, No selective outcome reporting, Other sources of bias; with “yes,” “no,” and “unclear” as evaluation results. Two independent authors will use Revman software to evaluate the quality of the selected articles. If there is a disagreement, a third author will participate in the discussion and ultimately determine the quality of the articles. The final results of the quality assessment will be presented in graph.

### 2.6 Data analysis

Authors will summarize the main information of the selected articles and any relevant question(s) according to the aim of this systematic review through Table 1; which include the study type, methods, description of population and relevant issues related to outcomes. If selected full-text articles are at least 7 or more, meta-analysis could be done after data extraction. If possible, a meta-analysis will be carried out using Stata15.0 software to compute pooled effect size (ES) with an estimate of 95% CI. If there are enough articles, heterogeneity test, subgroup analysis, sensitivity analysis, publication bias, and meta-regression will be performed.

#### 2.6.1 Heterogeneity test

Cochrane's *Q* test and Higgins *I*-squared statistic were undertaken to assess the heterogeneity of the included trials. A *P* < .10 or *I*^2^ > 50% suggested significant heterogeneity. Subgroup analysis, sensitivity analysis, and meta-regression were applied to analyze the origin of heterogeneity. RR is used as the effect size of binary variables and WMD/SMD is used as the effect size of continuous variables.

#### 2.6.2 Subgroup analysis

In subgroup analysis, the comparison will be based on clinical characteristics, such as article quality score, age, number of cautery, treatment time of cautery, follow-up time period after the course of treatment, etc. When the subgroup analysis is performed according to a certain clinical feature, each group are all homogeneous, and the combination is heterogeneous, indicating that the feature may be the source of heterogeneity.

#### 2.6.3 Sensitivity analysis

There are two methods used in sensitivity analysis, one is to change the analysis model and the other is to exclude articles one by one. An analysis model is chosen based on the *I*^2^ value. If *I*^2^ value is >50%, the random-effect model is used. If *I*^2^ is <50%, the fixed-effect model is used. When the articles are excluded one by one and there is a change in heterogeneity after removing one article, then this article may be the source of the heterogeneity.

#### 2.6.4 Meta-regression

When performing meta-regression analysis, the number of cautery, follow-up time period after the course of treatment, age, etc can be used as covariates to find the sources of heterogeneity. If *P* < .05, it means that the covariates are sources of heterogeneity.

#### 2.6.5 Publication bias

When ≥10 articles are included, Egger's Test or Begg's Test can be used for quantitative publication bias detection. If *P* > .05, it indicates that there is no publication bias. If *P* < .05, there is publication bias.

## 3 Discussion

Recurrent tonsillitis could serious influence one's physical and mental health, especially for children. The formation of tonsil biofilm and antibiotic resistance have brought difficulties in controlling the onset of the disease and complete cure of the disease.^[22,23]^ It is questionable whether after tonsillectomy would cause patient's immunity to be impaired and cause one to be more susceptible to respiratory infections or any serious illnesses.^[24–26]^ Therefore, there is an urgent to seek a treatment that cannot only preserve the tonsils but also control the recurrence of the disease. Cautery of traditional Chinese medicine can effectively control the number of attacks of RT, make it possible to reduce the rate of tonsillectomy, and allow one to reduce the use of antibiotics. This seems to provide a new program for the treatment of RT, but there is a lack of stronger evidence to prove it, so further research is needed.

The tonsils of patients with RT have abnormal local immune function.^[27]^ The inflammatory response may be mediated by the TLR2-MyD88-NF-κB signaling pathway.^[28]^ After the cautery method was performed, the study found that the normal ultrastructure can still be seen under the electron microscope.^[29]^ In addition, local fibrous tissue proliferation and changes in the number of immune cells and time phases can be seen.^[29]^ It has been observed that TCM cauterization may achieve anti-inflammatory effects by inhibiting the expression levels of IL-4, IL-5, IL-10, IFN-γ, and TGF-β and other cytokines in tonsil tissues.^[30]^ It was also found that the cautery method significantly increase the serum immunoglobulin level,^[31]^ and can inhibit the above signaling pathway. These may be the reasons for its effectiveness.

This research is expected to provide the following results. First, the effectiveness of the cautery method, the changes in the size of the tonsils, and the recurrence rate of tonsillitis will be used to evaluate the effectiveness of the treatment. Secondly, the safety of the method is evaluated based on the patients’ quality of life and the number of adverse events; thus, it provides some possible evidence for the treatment of RT. Furthermore, in the process of analysis, some shortcomings in the experimental design of TCM cautery treatment may be found, so as to provide some ideas for follow-up research.

## Author contributions

Sha Li, Hui Xie, Zhiqing Liu, and Han-Jen Chiang contributed to the study design, protocol development, and review by revising different versions. Jiao Liang, Lu Wang, and Qiu Wang were involved in the supervision, ensured the absence of errors and arbitrated in case of disagreement. Zhenzhen Han, Jiongke Li, and Yusi Li participated in the data collection, data analysis, and data management. Sha Li and Han-Jen Chiang engaged in the manuscript writing and data analysis. All authors have read and approved the final version of the manuscript.

**Conceptualization:** Hui Xie, Sha Li, Han-Jen Chiang, Zhiqing Liu.

**Data curation:** Zhenzhen Han, Jiongke Li, Yusi, Li.

**Investigation:** Jiao Liang, Lu Wang, Qiu Wang.

**Methodology:** Sha Li, Han-Jen Chiang.

**Resources:** Hui Xie, Sha Li.

**Supervision:** Jiao Liang, Lu Wang, Qiu Wang.

**Writing – original draft:** Sha Li, Han-Jen Chiang.

**Writing – review & editing:** Hui Xie, Sha Li, Han-Jen Chiang.

## Abbreviations

Abbreviations: PRISMA-P = Systematic Review and Meta-Analysis Protocols, RT = Recurrent tonsillitis, TCM = traditional Chinese medicine.
